# User perceptions of and willingness to pay for household container-based sanitation services: experience from Cap Haitien, Haiti

**DOI:** 10.1177/0956247815596522

**Published:** 2015-10

**Authors:** Kory Russel, Sebastien Tilmans, Sasha Kramer, Rachel Sklar, Daniel Tillias, Jennifer Davis

**Affiliations:** e-mail: kcrussel@stanford.edu; e-mail: stilmans@stanford.edu; e-mail: skramer@oursoil.org; e-mail: rsklar@berkeley.edu; e-mail: dtillias@future.edu; Department of Civil & Environmental Engineering, Stanford University, Via Ortega, Stanford, California USA; e-mail: jennadavis@stanford.edu

**Keywords:** container-based sanitation, faecal sludge management, Haiti, sanitation demand, urban sanitation, waterless sanitation

## Abstract

Household-level container-based sanitation (CBS) services may help address the persistent challenge of providing effective, affordable sanitation services for which low-income urban households are willing to pay. Little is known, however, about user perceptions of and demand for household CBS services. This study presents the results of a pilot CBS service programme in Cap Haitien, Haiti. One hundred and eighteen households were randomly selected to receive toilets and a twice-weekly collection service. After three months, changes in these households’ satisfaction with their sanitation situation, along with feelings of pride, modernity and personal safety, were compared to 248 households in two comparison cohorts. Following the service pilot, 71 per cent of participating households opted to continue with the container-based sanitation service as paying subscribers. The results from this study suggest that, in the context of urban Haiti, household CBS systems have the potential to satisfy many residents’ desire for safe, convenient and modern sanitation services.

**Figure:**
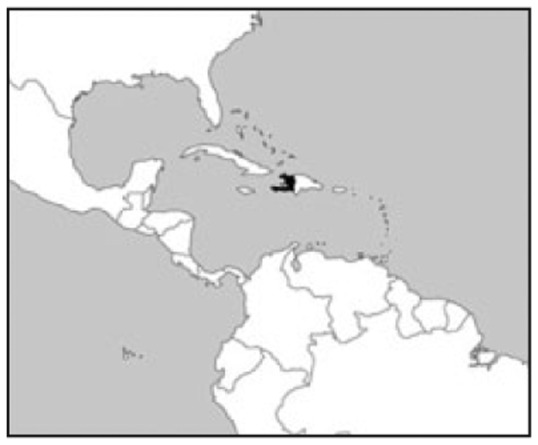


## I. Introduction

Effective isolation and removal of human waste is critical to protecting public health, particularly in dense urban environments. In low-income neighbourhoods of cities in developing countries, effective excreta management remains a persistent challenge. Traditional waterborne sewerage requires substantial financial resources and reliable supplies of piped water, and it is particularly appropriate for communities with accessible, gridded street patterns. Few, if any, of these conditions regularly exist in urban slums.^(1)^ On-site sanitation options, such as toilets with septic tanks or pit latrines, are the solutions most often adopted by those urban poor households with access to sanitation facilities.^(2)^ Whereas on-site facilities can provide a private place for defecation and initially isolate excreta (faeces and urine) from human contact, they too have limitations. The narrow, irregular street layouts of many poor urban neighbourhoods preclude emptying of septic tanks and latrine pits with suction trucks.^(3)^ Emptying is thus often undertaken manually, with the risk of exposure to faecal pathogens for both labourers and community members.^(4)^ Other options such as shared communal or public facilities are located at a distance from the home and are typically closed at night. Residents must therefore find other alternatives to meet their sanitation needs, and this can be particularly hazardous for women and children.^(5)^

Adding to these challenges is the limited public investment in slum sanitation infrastructure. Municipal governments are often reluctant to install long-lived assets in communities with uncertain or illegal tenure status.^(6)^ As a result, households themselves are largely responsible for financing, construction and maintenance of household sanitation facilities in slums. These responsibilities can be substantial, when one considers that a latrine:

1. represents a “lumpy” investment that requires a major up-front outlay of cash, or access to credit;
2. requires space, which can be hard to come by in dense urban communities; and
3. is a long-lived, immobile asset, and thus a risky investment for households that may experience relocation or eviction.

Many low-income city dwellers are also renters, understandably averse to investing in an asset that cannot be transported should they choose (or be forced) to relocate.^(7)^ Viewed from this perspective, the low effective demand for sanitation among developing country households that is consistently documented in the literature is perhaps not surprising.^(8)^

Container-based sanitation (CBS) has the potential to mitigate many of the challenges inherent in providing low-income urban households with reliable, effective sanitation services. A modern CBS system^(9)^ includes a toilet with a removable container that, when full, can be quickly exchanged for an empty container. The toilet design, along with the use of dry cover material, chemicals, or biodegradable plastic film, isolates wastes while eliminating odour and insects. All infrastructure associated with a CBS system – whether at the community or household scale – is typically situated above ground, reducing both construction costs and vulnerability to flooding. Excreta-filled containers can be sealed to reduce the likelihood of human contact with waste, then transported safely to a designated disposal or composting site. Water needs are limited to the amount required for anal cleansing and hand washing.

For the household subscribing, a CBS system would typically represent a substantially lower up-front investment (in some cases, a deposit equivalent to the monthly service fee) than would be the case for construction of a latrine or pour flush toilet. Clients would have access to their toilet day and night. What is less clear, however, is the extent to which households would value and be willing to pay for container-based sanitation services. Prior research suggests that households that are willing to pay for access to toilet facilities are not motivated by expected health gains, but instead by features such as convenience and privacy, as well as aspirations of enhanced pride and prestige.^(10)^ Because modern CBS services are currently offered in only a very small number of locations, we could find no published literature regarding their success in meeting users’ felt needs and preferences.

This paper contributes to filling this knowledge gap by presenting findings from a pilot project carried out in low-income neighbourhoods of Cap Haitien, Haiti. Over the course of three months, 141 households received a CBS service that included rental of an in-home toilet and twice-weekly collection of the waste. Our primary objectives were to measure changes in participating households’ perceptions and attitudes toward sanitation services before and after the CBS pilot, and to compare the magnitude of change among enrolled households with that of 248 comparison households. In addition, we evaluated willingness to pay for CBS service provision both before and after the pilot project. Finally, we assessed whether the introduction of a CBS service was associated with increased faecal contamination of the household environment, by monitoring the bacteriological quality of stored drinking water in a sub-sample of participating households.

This paper complements the analysis by Tilmans et al. of the same CBS pilot, published in the April 2015 issue of this journal.^(11)^ Whereas Tilmans et al. evaluate the extent to which the intervention increased the share of excreta produced in the community that was safely managed, our study focuses on the user experience with CBS, including the transition from a free pilot project to a paid subscription service.

## II. Study Site and Sample Frame

With a population of approximately 250,000, Cap Haitien, located on the country’s north coast, is the second largest city in Haiti. We could find no official estimates of sanitation service access in Cap Haitien, but across Haiti almost 8 per cent of the country’s 5.5 million urban residents are estimated to practise open defecation and fewer than one-third have their own toilet at home.^(12)^

The study reported here was undertaken in two Cap Haitien communities: the informal settlement of Shada and the formal settlement of Avyasyon.^(13)^ Together, these communities have a combined population of approximately 14,000 people. The treatment cohort (TC) – households that received the CBS toilet and service – and one comparison cohort (C1) were comprised of Shada households. The intervention was carried out in Shada in collaboration with SOIL, a non-governmental organization that had been operating public toilet facilities for several years.^(14)^ A second comparison cohort (C2) was comprised of households from the community of Avyasyon, located approximately two kilometres from Shada. High population density, irregular alley layout, and a high water table characterize both communities.

Households were recruited for the TC and C1 cohorts following preliminary meetings to discuss the research objectives with community leaders in Shada. The leaders then communicated with households in their constituencies about the study and their support for it. Households were selected for the treatment cohort in the following manner: 30 households were randomly selected from a previously compiled database of the approximately 1,600 households in Shada. For each of these households, the nearest nine neighbours were identified with geographic information system (GIS) software, using the shortest distances along alleyways or paths.^(15)^ Each of the 300 selected households was assigned a unique identification number, which was printed on a small slip of paper. For each cluster of 10 households, the relevant community leader drew the identification numbers at random out of a hat, determining the order in which households would be recruited for study enrolment. Study personnel then approached the first household selected in the cluster, carrying out informed consent procedures with a head of household. The process was repeated until five households in each cluster agreed to participate in the study.

Each potential respondent was asked whether his/her household had room for a private toilet; whether they would be interested in having a private toilet; and whether they would be willing to invest in improved sanitation. These screening questions were proposed by community leaders, who voiced concerns about households feeling pressure to participate even if they had no interest in upgrading their sanitation services. Approximately 23 per cent (n=70) of households selected for the treatment cohort were eliminated from the final sample; the most common reasons were a lack of space to accommodate a private toilet and tenants’ concerns about obtaining their landlords’ permission to participate. Another 89 households were selected for the TC but were never approached, because the target number of 141 participants had been met.

The comparison cohort in Shada was selected from the remaining ~1,300 households using a random number generator. Community leaders did not feel that it was necessary for them to participate in determining the order in which C1 households would be approached, since these participants would not receive a toilet or CBS services. Enumerators recruited comparison households in the order they were selected, until 153 households were recruited. Among the 205 households that were approached, 4 per cent (n=9) of households declined to participate; 21 per cent of the homes (n=43) were vacant or had no adult head of household available at the time of recruitment.

As no database of households in Avyasyon could be found, the comparison cohort in that community was selected using a systematic sampling methodology. Enumerators were assigned blocks of households delimited by GIS. Each enumerator started at the corner closest to the main road, soliciting every third household in a clockwise manner until s/he had returned to the first household interviewed on the block. A total of 218 households were approached and solicitation continued until 151 households were recruited. Approximately 7 per cent (n=15) of households approached declined to participate; another 24 per cent of homes (n=52) were vacant or had no head of household available during recruitment.

For all cohorts, three attempts were made to recruit a selected household before replacing it with another in the same community. If the head of household was at his or her place of employment, a time was set for the enumerator to return and administer the survey when s/he was available. The free and informed consent of each participant was obtained, and both the Institutional Review Board of Stanford University, California, USA and the National Bioethics Board of Haiti approved the study protocol. Additionally, the research protocol and CBS intervention were reviewed and approved by the Haitian Water and Sanitation Directorate (DINEPA) at both the local and national administrative levels.

Twenty-three of the 141 households selected for the treatment cohort completed a baseline interview but subsequently dropped out of the study. The Shada comparison cohort (C1) enrolled 153 initially but lost 36 following the baseline phase. The Avyasyon cohort (C2) began with 151 households; 20 dropped out of the study between baseline and endline. A complete attrition analysis is provided in the supporting information section (see this paper’s supplementary material online).

## III. Sanitation Intervention

User-centric design was employed to develop the toilet used in this study. Research team members conducted semi-structured interviews with a variety of potential users and focus group members to gain a better understanding of residents’ lifestyles and aspirations. Insights from these activities included the fact that a private toilet is a symbol of wealth, luxury and cleanliness for households in these communities. Flush toilets are referred to as *konfò modèn*, which translates to “modern comfort”. Such toilets are believed not only to rid households of waste, odours and flies, but also to represent the essence of upper-class life. Findings from this formative research were incorporated into the development of several prototype toilets, each of which was tested by seven households for approximately one month.

**Photo 1:**
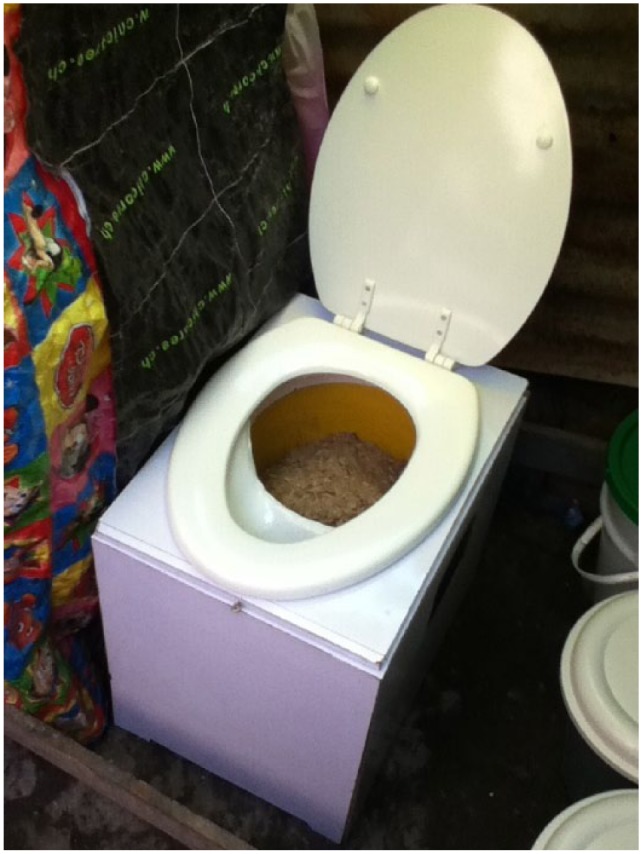
CBS toilet used in study © Sebastien Tilmans (2012).

Iterative testing continued until a design appropriate for informal settlements in Cap Haitien had been achieved. The toilet used in this study separates urine and faeces for collection in two different containers. The containers are housed in a protective box that has a traditional toilet seat mounted on its top.^(16)^ No superstructure was provided for the toilet, and placement within the household was left to the discretion of users (Photo 1).

Each household in the treatment cohort received a toilet, a second collection container filled with dry cover material (crushed peanut shells and dry sugarcane bagasse, referred to as Bonzodè), and twice-weekly collection service, which included replenishment of the Bonzodè. Households were also provided with oral instructions and demonstrations on the correct use of the toilet. They were instructed to scatter a handful of Bonzodè over the faeces in the container after every use, similar to methods used in composting toilets. Households were also responsible for disposing of collected urine, in drainage canals, the sea, public toilets or soakaway pits, once the one-gallon container was full. Twice weekly, a technician employed and trained by SOIL collected each household’s faeces container, sealed its lid and transported it on a handcart to a pickup truck. Containers were then transferred to a centralized compost facility where they were emptied and cleaned. Additional details about these procedures can be found in Preneta et al.^(14)^ and Tilmans et al.^(17)^

Each treatment cohort household signed a form acknowledging that the three-month study was a free pilot, and that those wishing to continue with the CBS service subsequently would have to subscribe at a rate of US$ 5/month/household.^(18)^ If the head of household could not read or write, a trusted friend or family member was permitted to sign the document in his/her stead. Households were also informed that that they could terminate the CBS service at any time and they were not obligated to become a paying subscriber once the pilot had concluded. Households in the two comparison cohorts received no toilet, training, or waste collection service from the study team during the three-month study period. The CBS system was not discussed prior to the baseline survey with comparison cohort members, although those residing in Shada were able to become subscribing customers upon conclusion of the pilot.

During data analysis, a household was defined as a member of the treatment cohort (TC) if it was interviewed at baseline, received a toilet from the study team, and was interviewed at endline. A household was considered a member of the comparison cohort if it was interviewed at baseline, did not receive a toilet from the study team, and was interviewed at endline. Thus, any household that completed only one interview (either baseline or endline) was excluded from analysis. In addition, two C1 households received CBS toilets from TC households that had left the study. Taking the most conservative approach, we classified these C1 households as C1 respondents during data analysis.

## IV. Data Collection and Analysis Methods

In-person interviews with a male or female head of household were conducted with all participating households before the intervention began (baseline) and three months later, at the end of the free trial period (endline). All data collection occurred during dry months (October and February). The survey was coded for use on personal digital assistants (PDAs) using the software package The Survey System (Creative Research Systems, Petaluma, CA, USA). The baseline survey was developed, translated and pre-tested over the course of several months. The endline survey included many of the same questions as the baseline survey, but also included a module that assessed treatment households’ experience with the CBS toilet and service. All surveys were administered in Haitian Creole and took a median of 43 minutes to complete.

Samples of stored drinking water were collected from a sub-sample of 172 households at baseline and 121 households at endline. Each respondent was asked to extract water from the storage container as s/he normally would and to pour it into the sampling vessel. All water samples were tested for the presence of chlorine using SenSafe® Free Chlorine Water Check test strips (Industrial Test Systems, Inc., Rock Hill, SC, USA). If chlorine was detected, it was neutralized with sodium thiosulfate in a Whirl-Pak® Thio-Bag® (NASCO Corp., Fort Atkinson, WI, USA).

Water samples were placed on ice and transported to a field laboratory for processing within six hours of collection. All samples were processed using Colilert®-18 (IDEXX Laboratories, Inc., One IDEXX Drive, Westbrook, ME, USA) and Quanti-Tray®/2000 to generate most probable number (MPN) colony counts of the faecal indicator bacteria *E. coli*. Volumes of 100 mL were processed, providing lower and upper detection limits of 1 and 2,400 MPN/100 millilitres, respectively. If no fluorescence was visible in the tray, then a value of 0.5 MPN was assigned. If all wells were positive, the value of the upper detection limit was assigned to the tray. A total of 148 baseline and 115 endline samples were successfully processed in accordance with the study protocol. A negative control and duplicate household sample were taken in the field each day as quality control measures.

Six Haitian nursing students were selected to be enumerators and received intensive training over a two-week period in interviewing skills, the objectives and content of the household survey, sterile water sampling techniques and the use of PDAs for data collection. A one-week refresher course was administered prior to the endline survey, three months after the baseline.

Analysis of variance (ANOVA) with post-hoc tests was used to compare cohort-level characteristics at baseline, as well as to compare characteristics of households that completed the study to those that dropped out following the baseline. Changes in reported attitudes toward sanitation services over the course of the intervention were analysed using a repeated measures binomial logit model. Cohort assignment was treated as a fixed effect, and the analysis included a random intercept to account for baseline differences across cohorts. Non-normally distributed continuous variables (e.g., *E. coli* MPNs) were log transformed. ANOVA with post-hoc tests was also used to compare cohort-level differences in the mean of log-transformed *E. coli* MPN values at baseline and endline. All statistical analyses were performed with SPSS Statistics 22.0 (SPSS Inc., Chicago, Illinois, USA).

## V. Results

### a. Household characteristics

Information collected from participants in all three cohorts at baseline was pooled and compared with a nationally representative Demographic and Household Survey (DHS) completed in 2012 with funding from the United States Agency for International Development.^(19)^ Overall, households in the study sample have a socioeconomic and demographic profile that is similar to that of urban households in Haiti as a whole. The average household size in our sample was 6.0 persons. Eighty per cent of sample households owned at least one mobile phone, and 40 per cent owned a radio. The DHS survey found an average urban household size of 5.8 persons in Haiti. Mobile phone and radio ownership was reported as 77 per cent and 55 per cent, respectively. Educational attainment was similar between the CBS study and DHS samples, with roughly 40 per cent of household heads in each group having completed some primary school and 30 per cent having completed some secondary education. A smaller share of study households (32 per cent) had electricity service in their homes as compared to the DHS sample (59 per cent). In addition, 40 per cent of study participants reported practising open defecation or using flying toilets,^(20)^ compared to only 11.5 per cent of DHS respondents.

Comparing household characteristics across the three study cohorts, we observed no statistically significant differences between households in the treatment cohort and the comparison cohort from Shada (C1) across a variety of socioeconomic and demographic variables (Table 1). The Avyasyon comparison cohort (C2) included larger households and a higher share of homeowners than either the treatment or C1 cohort (all p<0.01).

**Table 1:** Baseline characteristics of households that completed entire study, by cohort

	Treatment cohort	Comparison cohort 1	Comparison cohort 2
	(N=117–118)^(a)^	(N=116–117)	(N=130–131)
Mean (standard deviation) household size	5.5 (2.7)	5.8 (2.8)	6.7^(b,c)^ (2.7)
Mean (standard deviation) respondent age	37.5 (13.0)	35.3 (12.5)	37.7 (12.0)
Mean (standard deviation) reported monthly expenditure (US$)	183.03 (154.01)	198.28 (182.64)	135.79 (301.85)
Median reported monthly expenditure (US$)	151.80	140.84	82.07^(d)^
% female respondents	65	71	70
% homeowners	62	62	89^(b,c)^
% with electricity	33	35	27
% attended some primary school	44	41	38
% attended some secondary school	30	30	29
% with a corrugated tin roof	79	79	82
% owning a television	42	32	33
% owning a mobile phone	76	77	87
% using own or a neighbour’s private latrine^(e)^	28	28	50^(b,c)^
% using public toilets^(e)^	51	52	5^(b,c)^
% practising open defecation or using flying toilets^(e)^	40	35	45

NOTES:

(a) Sample sizes vary by analysis because of missing data or non-response.

(b) Mean is significantly different from that of treatment cohort (ANOVA, Tukey’s post-hoc test, p < 0.01).

(c) Mean is significantly different from that of comparison cohort 1 (ANOVA, Tukey’s post-hoc test, p < 0.01).

(d) Median is significantly different from those of treatment cohort and comparison cohort 1 (Mood’s median test, p < 0.01).

(e) Multiple responses permitted for each household.

### b. Sanitation practices and attitudes toward sanitation services

At baseline, the sanitation practices reported by households in the treatment and C1 cohorts were relatively similar (Table 1). Twenty-eight per cent were using their own or a neighbour’s private latrine, and roughly half were relying on public toilets. By contrast, a significantly higher share of households in the C2 cohort reported using private latrines, and only 5 per cent were making use of public toilets (both p<0.01). Between 35 per cent (C1) and 45 per cent (C2) of households in each cohort reported practising open defecation and/or using flying toilets, but the difference is not statistically significant.

At both baseline and endline, respondents were asked to rate their satisfaction with their households’ overall sanitation situation on a four-point scale that included the options “very satisfied”, “generally satisfied”, “generally dissatisfied” and “very unsatisfied”.^(21)^ For analysis purposes, responses were binned into a binary variable of “satisfied” or “dissatisfied”. A repeated measures logistic regression model was used with control variables for the respondent’s age and gender; whether the respondent had completed primary school; per-capita daily household expenditure on food, transport, services, clothing, etc.; and the number of people in the household. At endline, a TC household was 16.7 and 9.5 times more likely to report satisfaction with its sanitation situation, on average, than a C1 or C2 household, respectively (Table 2).

**Table 2:** Changes in perceptions of sanitation situation between baseline and endline, by cohort: repeated measures logistic regression model

Survey prompt: “Overall, how satisfied are members of your household with your current sanitation situation?”					
		% at baseline	% at endline	OR^(a)^ (95% CI)^(b)^	P-value
**“very” or “generally” satisfied**	Treatment (N=116)^(c)^	32	87		
	Comparison 1 (N=116)	39	35	OR=16.7 (6.9–40.0)	<0.001
	Comparison 2 (N=129)	26	36	OR=9.5 (4.0–22.7)	<0.001
Survey prompt: “To what extent do you agree or disagree with the following statement? ‘My household’s current sanitation condition makes me feel…’”					
**… proud**	Treatment (N=89)	25	94		
	Comparison 1 (N=110)	34	29	OR=66.7 (21.7–200.0)	<0.001
	Comparison 2 (N=129)	25	33	OR=41.7 (13.5–125.0)	<0.001
**… modern**	Treatment (N=103)	17	72		
	Comparison 1 (N=110)	29	13	OR=35.7 (13.9–90.9)	<0.001
	Comparison 2 (N=130)	22	24	OR=14.3 (5.7–35.7)	<0.001
**… ashamed**	Treatment (N=103)	53	6		
	Comparison 1 (N=110)	46	65	OR=0.02 (0.01–0.06)	<0.001
	Comparison 2 (N=129)	66	51	OR=0.09 (0.03–0.27)	<0.001
**… safe**	Treatment (N=103)	32	92		
	Comparison 1 (N=110)	48	35	OR=55.5 (20.4–142.9)	<0.001
	Comparison 2 (N=129)	34	40	OR=23.8 (8.9–62.5)	<0.001
Survey prompt: “Would you say that yours is a household that others in Shada respect…”					
**“…a great deal?” or “…more than average?”**	Treatment (N=111)	63	68		
	Comparison 1 (N=113)	77	62	OR=2.7 (1.2–6.1)	0.02
	Comparison 2 (N=125)	74	65	OR=1.9 (0.9–4.1)	0.11

NOTES:

(a) OR = odds ratio.

(b) CI = confidence interval. Lower and upper confidence interval bounds are in parentheses.

(c) Sample sizes vary by analysis because of missing data or non-response.

Each respondent was also asked to what degree s/he agreed with a series of statements, each with the leading prompt “My household’s current sanitation condition makes me feel…” Responses were elicited regarding feelings of pride, modernity, shame and safety. Answer options included “Strongly agree”, “Generally agree”, “Generally disagree” and “Strongly disagree.” In all cases, a respondent in the treatment cohort was significantly more likely than one in either comparison cohort to report, at endline, that his/her household’s sanitation situation conferred feelings of pride, modernity and safety (all p<0.001). S/he was also significantly less likely to report feeling ashamed of his/her household’s sanitation situation (p<0.001 for comparisons of both C1 and C2 with TC).

Finally, respondents were asked how much they felt that other households in their community respected their household. Answer options included “A great deal”, “Somewhat more than the average household”, “Somewhat less than the average household” and “Little or not at all.” For this question, treatment cohort respondents were significantly more likely than C1 respondents to report that they felt their households were highly respected (p=0.02) but not significantly more likely than C2 respondents. Notably, this question was asked without explicit reference to sanitation services or practices.

Whereas all of the estimated odds ratios are characterized by considerable uncertainty, those for the Shada comparison group (C1) are consistently greater than those for households in the Avyasyon comparison group (C2). For example, at endline a TC respondent was 55.5 times more likely than a C1 respondent to say that his/her household’s sanitation services conferred feelings of safety, and 24 times more likely than a C2 respondent.

### c. User demand

The endline survey also included a module designed to assess respondents’ willingness to pay for the CBS toilet and waste collection service. Treatment cohort households had first-hand experience with the service, and some C1 cohort households had become familiar through interactions with neighbours participating in the pilot. The survey administered to comparison cohort households included a detailed description of the toilet and collection service. Each respondent was asked whether his/her household would be willing and able to pay a monthly price of US$ 5 or US$ 7.50 – values randomly assigned by the PDA during each interview – for the CBS service.

Among the respondents in the treatment cohort asked about the US $5 fee, 77 per cent said they were “very” or “somewhat likely” to pay this amount each month for CBS services. A similar percentage of comparison cohort respondents (73 per cent of C1 and 74 per cent of C2) reported a willingness and ability to pay this amount. Forty-nine per cent of respondents asked about their willingness to pay US$ 7.50 in each cohort said they would be willing to pay this amount. The overall mean monthly willingness to pay, assuming a value of zero for respondents who gave both “no” and “don’t know” answers, was US$ 3.58. Respondents who agreed to either price were asked why their households would be interested in subscribing to CBS services. Three-quarters cited the convenience of a household toilet and collection service. A smaller percentage mentioned improved health (8 per cent), greater personal safety (4 per cent), or lower costs compared with their current practices (3 per cent).

Data on actual household decision-making at the conclusion of the free pilot generally accord with these willingness-to-pay survey results. At the conclusion of the pilot programme in February 2013, 127 households were using the CBS toilet and service.^(22)^ Nine months later, in November 2013, 90 (71 per cent) of those households continued to use the fee-based service. During this period, on-time payment rates were at least 80 per cent. Since that time, SOIL has expanded the fee-based service to more than 300 households. For several months in late 2013 and early 2014, the limited enforcement of toilet repossession from delinquent clients caused a decline in on-time payment rates. Since that time, renewed enforcement efforts have brought payment collection rates back to 60 per cent, with continuing improvement.

### d. Stored water contamination

Given the small number of water samples processed, it was not possible to estimate a multivariate regression model to evaluate changes in mean *E. coli* concentrations across cohorts. Using analysis of variance (ANOVA) with Tukey’s post-hoc test, we compared mean log-transformed *E. coli* concentrations between cohorts at baseline and again at endline. At baseline, the mean contamination level of stored water in the treatment cohort was not significantly different than that of either comparison cohort; however, the value for the C2 cohort was significantly lower than that of the C1 cohort (Table 3). At endline, none of the observed differences between cohorts was statistically significant (all p>0.10). For all cohorts, the log mean concentration was lower during endline sampling as compared to baseline. More specifically, log mean concentration values were 42 per cent and 40 per cent lower for the C1 and C2 cohorts, respectively, and 75 per cent lower for the treatment cohort (Table 3).

**Table 3:** Mean (standard deviation) of log-transformed most probable number/100 mL of *E. coli* in stored drinking water

	Baseline	Endline
**Treatment cohort (N = 35, 32)^(a)^**	0.8 (1.3)^(b)^	0.2 (0.9)
**Comparison cohort 1 (N = 47, 30)**	1.2 (1.2)	0.7 (1.1)
**Comparison cohort 2 (N = 66, 53)**	0.5^(c)^ (0.9)	0.3 (0.9)

NOTES:

(a) Sample sizes vary by analysis because of missing data or non-response.

(b) Standard deviations are in parentheses.

(c) Mean is significantly different from that of comparison cohort 1 at baseline (ANOVA, Tukey’s post-hoc test, p < 0.01).

## VI. Discussion

Container-based sanitation systems could in theory address many of the technical, financial and political challenges of making long-term sanitation infrastructure investments in low-income urban communities. The results from this study suggest that, in the context of urban Haiti, household CBS systems have the potential to satisfy many residents’ desire for safe, convenient and modern sanitation services. In this setting, it appears that well-designed toilets and professionalized collection service procedures can also avoid the stigma historically associated with bucket latrines and similar “low-tech” options. In addition, while recognizing that our study did not have the statistical power to evaluate the magnitude of change in stored water contamination or incidence of diarrhoeal disease, none of the collected data suggests that introduction of the CBS service was associated with increased faecal contamination of the household environment. Additional research that quantifies such public health impacts of CBS service provision would be a valuable contribution.

It is important to note that many of the data analysed in this study were self-reported by participants, which raises the possibility of both social desirability and strategic bias. Steps were taken to prevent each household in the TC and C1 cohorts from learning whether it would receive a CBS toilet from the study team; however, it is possible that some participants obtained such information. If so, this knowledge could have influenced respondents’ answers to questions in the baseline survey. Additionally, this study is vulnerable to courtesy bias, given that members of the treatment cohort received access to an in-home sanitation facility free of charge for a three-month period. Absent corroborating data, we would suspect an upward bias in reports of the CBS toilet use, as well as of households’ satisfaction with the service. Our findings are, however, supported by revealed preference data in the form of study participants opting to become paying subscribers during the post-pilot period. Reported toilet use rates are also consistent with waste collection data as reported by Tilmans et al.^(23)^

We also note that, while satisfaction and positive self-assessments increased among treatment cohort households following deployment of the CBS toilets, those of the nearby C1 households decreased markedly. These findings could be interpreted to mean that the intervention exacted a socio-psychological cost on Shada households that did not receive a CBS toilet. Taken by itself, this outcome could be viewed as undesirable. At the same time, such generation of shame, pride and social pressure is precisely what is believed to motivate behaviour change in programmes such as community-led total sanitation.^(24)^ Traditional marketing also employs such strategies to catalyse action among consumers, who compare themselves to peers or to an idealized version of themselves. Of course, with respect to motivating improvements in sanitation practices, it is important not simply to create demand but to offer alternatives that households want and can feasibly obtain.^(25)^

Prior research suggests that the attitudinal shifts documented among treatment cohort households between baseline and endline are good indicators of effective demand for CBS services. Several studies have found associations among awareness of, perceived convenience of, and level of satisfaction with a sanitation solution and the likelihood of adopting it.^(26)^ In Shada, 71 per cent of households in the treatment cohort transitioned from being free pilot participants to being paying customers. This conversion rate is particularly encouraging given the challenges of promoting household investment in sanitation.^(27)^ At US$ 5 per month, the CBS service in Shada represents 2.9 per cent of the mean monthly expenditure for sample households. To put that figure in perspective, households reported spending more than twice that amount each month for mobile phone credit (mean of US$ 11.42, or 6.6 per cent of total expenditure), and for water supply (US$11.98, or 6.9 per cent). Nevertheless, for the almost 30 per cent of treatment cohort households that did not transition to being paying CBS subscribers, cost was the most commonly cited barrier. In addition, as described in Tilmans et al.,^(28)^ the US$ 5 fee does not fully cover the costs of service provision at this pilot scale. Thus, while this study suggests potential for CBS service to reach hard-to-serve communities, it underscores the challenge of financing sanitation services solely through user fees in such contexts.

This study also highlights the difficulty of providing household-level sanitation services in densely populated communities. As noted in Section II, almost one-third of households selected for the pilot were eliminated during screening because of space limitations in their homes. We thus see a need for additional innovation in CBS toilet and service design that addresses extreme space constraints. Future work might consider adapting the CBS model to serve a small number of adjacent households that are unable to accommodate individual toilets. Given the aspirational nature of private, exclusive service – as well as mounting evidence that the lack of household sanitation may be an important contributor to physical and sexual assault of women – it will be important to ensure that shared facilities are safely accessible both day and night.^(29)^

The findings from this study suggest that, within the menu of options for sanitation service provision, CBS systems may be useful for reaching low-income households residing in dense, unregularized urban communities. Much more needs to be learned about the socioeconomic, cultural and geographic contexts in which CBS services are likely to be more or less effective, and thus about the size of the potential market for this approach. Similarly, additional efforts are needed to identify the adaptations needed – both to toilet and waste conveyance technologies, as well as to the CBS service delivery business model – in order to meet users’ needs in different settings. Finally, we note that scaling sanitation innovations beyond the pilot phase can be impeded when relevant regulatory frameworks are absent, incomplete or contradictory. Future work that deals explicitly with such institutional considerations of scaling container-based sanitation services would thus be a valuable contribution.

## Supplementary Material

Supplementary material

## Footnotes

1. The term “slum” often has derogatory connotations and can suggest that a settlement needs replacement or can legitimate the eviction of its residents. However, it is a difficult term to avoid for at least three reasons. First, some networks of neighbourhood organizations choose to identify themselves with a positive use of the term, partly to neutralize these negative connotations; one of the most successful is the National Slum Dwellers Federation in India. Second, the only global estimates for housing deficiencies, collected by the United Nations, are for what they term “slums”. And third, in some nations, there are advantages for residents of informal settlements if their settlement is recognized officially as a “slum”; indeed, the residents may lobby to get their settlement classified as a “notified slum”. Where the term is used in this journal, it refers to settlements characterized by at least some of the following features: a lack of formal recognition on the part of local government of the settlement and its residents; the absence of secure tenure for residents; inadequacies in provision for infrastructure and services; overcrowded and sub-standard dwellings; and location on land less than suitable for occupation. For a discussion of more precise ways to classify the range of housing sub-markets through which those with limited incomes buy, rent or build accommodation, see *Environment and Urbanization* Vol 1, No 2 (1989), available at http://eau.sagepub.com/content/1/2.toc.

2. As of 2012, more than 100 million people living in urban areas reported having no regular access to a sanitation facility, i.e., practising open defecation. Joint Monitoring Programme (2014), *Progress on Drinking Water and Sanitation: 2014 Update*, WHO and UNICEF, 28 pages.

3. Wegelin-Schuringa, M and T Kodo (1997), “Tenancy and sanitation provision in informal settlements in Nairobi: revisiting the public latrine option”, *Environment and Urbanization* Vol 9, No 2, pages 181–190.

4. Adubofour, K, K Obiri-Danso and C Quansah (2013), “Sanitation survey of two urban slum Muslim communities in the Kumasi metropolis, Ghana”, *Environment and Urbanization* Vol 25, No 1, pages 189–207; also Joshi D, B Fawcett and F Mannan (2011), “Health, hygiene and appropriate sanitation: experiences and perceptions of the urban poor”, *Environment and Urbanization* Vol 23, No 1, pages 91–111.

5. See reference 4, Joshi et al. (2011); also Bapat, M and I Agarwal (2003), “Our needs, our priorities; women and men from the slums in Mumbai and Pune talk about their needs for water and sanitation”, *Environment and Urbanization* Vol 15, No 2, pages 71–86.

6. See reference 4, Adubofour et al. (2013) and Joshi et al. (2011); also McFarlane, C (2008), “Sanitation in Mumbai’s informal settlements: state, ‘slum’, and infrastructure”, *Environment and Planning A* Vol 40, No 1, pages 88–107.

7. Isunju, J B, K Schwartz, M A Schouten, W P Johnson and M P van Dijk (2011), “Socio-economic aspects of improved sanitation in slums: A review”, *Public Health* Vol 125, No 6, pages 368–376; also Okurut, K and K J Charles (2014), “Household demand for sanitation improvements in low-income informal settlements: A case of East African cities”, *Habitat International* Vol 44, pages 332–338; and Cairncross, S (1992), *Sanitation and water supply: practical lessons from the decade*, World Bank, 63 pages.

8. See reference 4, Joshi et al. (2011); see also reference 7, Isunju et al. (2011) and Okurut and Charles (2014); Jenkins, M W and S Sugden (2006), *Rethinking sanitation: Lessons and innovation for sustainability and success in the new millennium*, Human Development Report Office, United Nations Development Programme, 37 pages; and Briscoe, J (1996), “Financing water and sanitation services: the old and new challenges”, *Water Supply* Vol 14, No 3, pages 1–17.

9. We use the term “modern CBS system” to distinguish this model from “bucket latrines” that are ineffective in isolating waste and are generally perceived as unpleasant to use. Nilsson, D (2006), “A heritage of unsustainability? Reviewing the origin of the large-scale water and sanitation system in Kampala, Uganda”, *Environment and Urbanization* Vol 18, No 2, pages 369–385; also Letema S, B van Vliet and J B van Lier (2014), “Sanitation policy and spatial planning in urban East Africa: Diverging sanitation spaces and actor arrangements in Kampala and Kisumu”, *Cities* Vol 36, pages 1–9.

10. See reference 7, Isunju et al. (2011); also Jenkins, M W and V Curtis (2005), “Achieving the ‘good life’: Why some people want latrines in rural Benin”, *Social Science & Medicine* Vol 61, No 11, pages 2446–2459; Jenkins, M W and S Cairncross (2010), “Modelling latrine diffusion in Benin: towards a community typology of demand for improved sanitation in developing countries”, *Journal of Water and Health* Vol 8, No 1, pages 166–183; and Black, M and B Fawcett (2010), *The Last Taboo*, Routledge, 272 pages.

11. Tilmans, S, K Russel, R Sklar, L Page, S Kramer and J Davis (2015), “Container-Based Sanitation: Assessing Costs and Effectiveness of Excreta Management in Cap Haitien, Haiti”, *Environment and Urbanization* Vol 27, No 1, pages 89–104.

12. See reference 2, Joint Monitoring Programme (2014); also Arias, D, J, J Leguía and A Sy (2013), *Determinants of Agricultural Extension Services*, LCSSD Food Paper Series, 34 pages.

13. The UN Human Settlements Programme (UN-HABITAT) defines informal settlements as: i) residential areas where a group of housing units has been constructed on land to which the occupants have no legal claim, or which they occupy illegally; and ii) unplanned settlements and areas where housing is not in compliance with current planning and building regulations (unauthorized housing). UN-HABITAT (2006), *Enabling shelter strategies: Review of experience from two decades of implementation*, Nairobi, 265 pages.

14. Preneta, N, S Kramer, B Magloire and J M Noel (2013), “Thermophilic co-composting of human wastes in Haiti”, *Journal of Water, Sanitation and Hygiene for Development* Vol 3, No 4, pages 649–654.

15. Ho, J C, K C Russel and J Davis (2014), “The challenge of global water access monitoring: evaluating straight-line distance versus self-reported travel time among rural households in Mozambique”, *Journal of Water and Health* Vol 12, No 1, pages 173–183.

16. See reference 11.

17. See references 11 and 14.

18. The US$ 5 price included rental of the toilet, collection service and supply of Bonzodè. This price did not cover the full costs of service provision at the pilot scale. See reference 11 for a detailed analysis of service costs.

19. Ministry of Public Health and Population [*le Ministère de la Santé Publique and de la Population*] (MSPP), Haitian Childhood Institute [*l’Institut Haïtien de l’Enfance*] (IHE) and ICF International (2013), *2012 Haïti Mortality, Morbidity, and Service Utilization Survey: Key Findings*, Calverton, Maryland, 24 pages.

20. A “flying toilet” is a plastic bag that is used to collect faeces during in-home defecation and is then thrown into an alley, waterway, solid waste bin, or rooftop.

21. “Don’t know” responses were also permitted, but the option was not prompted and was selected by no more than two respondents in each study phase. Respondents who provided “Don’t know” answers were not included in multivariate statistical analyses.

22. This number includes the 118 households that completed the study, plus an additional nine that received a toilet at baseline but did not complete a baseline interview. See reference 11.

23. See reference 11.

24. Pattanayak, S K, J-C Yang, K L Dickinson, C Poulos, S R Patil, R K Mallick, J L Blitstein et al. (2009), “Shame or subsidy revisited: social mobilization for sanitation in Orissa, India”, *Bulletin of the World Health Organization* Vol 87, No 8, pages 580–587; also Patil, S R, B F Arnold, A L Salvatore, B Briceno, S Ganguly, J M Colford and P J Gertler (2014), “The Effect of India’s Total Sanitation Campaign on Defecation Behaviors and Child Health in Rural Madhya Pradesh: A Cluster Randomized Controlled Trial”, *PLoS Medicine* Vol 11, No 8, page e1001709.

25. Bartram, J, K Charles, B Evans, L O’Hanlon and S Pedley (2012), “Commentary on community-led total sanitation and human rights: should the right to community-wide health be won at the cost of individual rights?”, *Journal of Water and Health* Vol 10, No 4, pages 499–503.

26. See reference 7, Isunju et al. (2011) and Okurut and Charles (2014); also Jenkins, M W and B Scott (2007), “Behavioral indicators of household decision-making and demand for sanitation and potential gains from social marketing in Ghana”, *Social Science & Medicine* Vol 64, No 12, pages 2427–2442.

27. See reference 4, Joshi et al. (2011); see also reference 7, Isunju et al. (2011) and Okurut and Charles (2014); and reference 8, Jenkins and Sugden (2006) and Briscoe (1996).

28. See reference 11.

29. See reference 5, Bapat and Agarwal (2003); also Sommer, M, S Ferron, S Cavill and S House (2015), “Violence, gender and WASH: spurring action on a complex, under-documented and sensitive topic”, *Environment and Urbanization* Vol 27, No 1, pages 105–116.
