# Supplementary material for: User perceptions of and willingness to pay for household container-based sanitation services: experience from Cap Haitien, Haiti
Source: Environ Urban. 2015 Oct;27(2):525–40. doi: 10.1177/0956247815596522 (PMC4645720; doi:10.1177/0956247815596522)
Supplement: Supplementary material [file Russel_supplementary_material.pdf]

User perceptions of and willingness to pay for household container-based sanitation services: experience from Cap Haitien, Haiti

Supporting information

Attrition analysis results

An attrition analysis found that households that dropped out of the study were less likely to own their home, or to have a mobile phone, TV, radio or access to electricity, as compared to households that completed both baseline and endline interviews (Table S1). No significant difference in the amount of rent, monthly expenditures, or household size was observed between the two groups. Dropout households were found to be significantly more likely than those that completed the study to use public toilets (as opposed to practising open defecation or using flying toilets). There was no significant difference, however, in the percentage of households in each group that used their own or a neighbour’s private latrine.

TABLE S1
Characteristics of households that completed full study versus those who left following baseline interview

**Household/respondent characteristic**	**Households that completed baseline & endline interviews**	**Households that completed baseline interviews only**
Mean household size (N=363, 79)	6.0 (2.8)	5.5 (3.2)
Mean respondent age (N=358, 78)	36.8 (12.5)	38.0 (14.6)[a]
Mean monthly rent (US$) (N=103, 38)	11.77 (6.73)	10.98 (8.71)
Mean reported monthly expenditure (US$) (N=366, 79)	171.00 (227.19)	173.13 (205.94)
% respondents who were female (N= 365, 79)	69	66
% home owners (N= 359, 77)	71	66
% with electricity (N= 364, 79)	32	20[b]
% respondents who had attended some primary school (N= 361, 78)	41	37
% respondents who had attended some secondary school (N= 361, 78)	29	35
% using cement or blocks for house (N= 366, 79)	96	97
% using corrugated tin for roof (N= 364, 79)	20	25[a]
% owning a television (N= 364, 79)	36	28[a]
% owning a mobile phone (N= 362, 79)	80	67[a]
% owning a radio (N= 364, 79)	40	34[b]
% using a neighbour's or own private latrines (N= 366, 79)	36	33
% using public toilets (N= 366, 779)	35	51[b]
% using open defecation or flying toilets (N= 366, 79)	40	30[b]

NOTES: Standard deviation values for the second and third columns are in parentheses. Sample sizes vary by analysis because of missing data and "Don't know" responses.
[a]Mean is significantly different from that of the participants that completed the study (t-test, $p < 0.05$).
[b]Mean is significantly different from that of the participants that completed the study (t-test, $p < 0.00$).
